# Supplementary figures and images for: Niche Partitioning between Coastal and Offshore Shelf Waters Results in Differential Expression of Alkane and Polycyclic Aromatic Hydrocarbon Catabolic Pathways
Source: mSystems. 2020 Aug 25;5(4):e00668-20. doi: 10.1128/mSystems.00668-20 (PMC7449609; doi:10.1128/mSystems.00668-20)

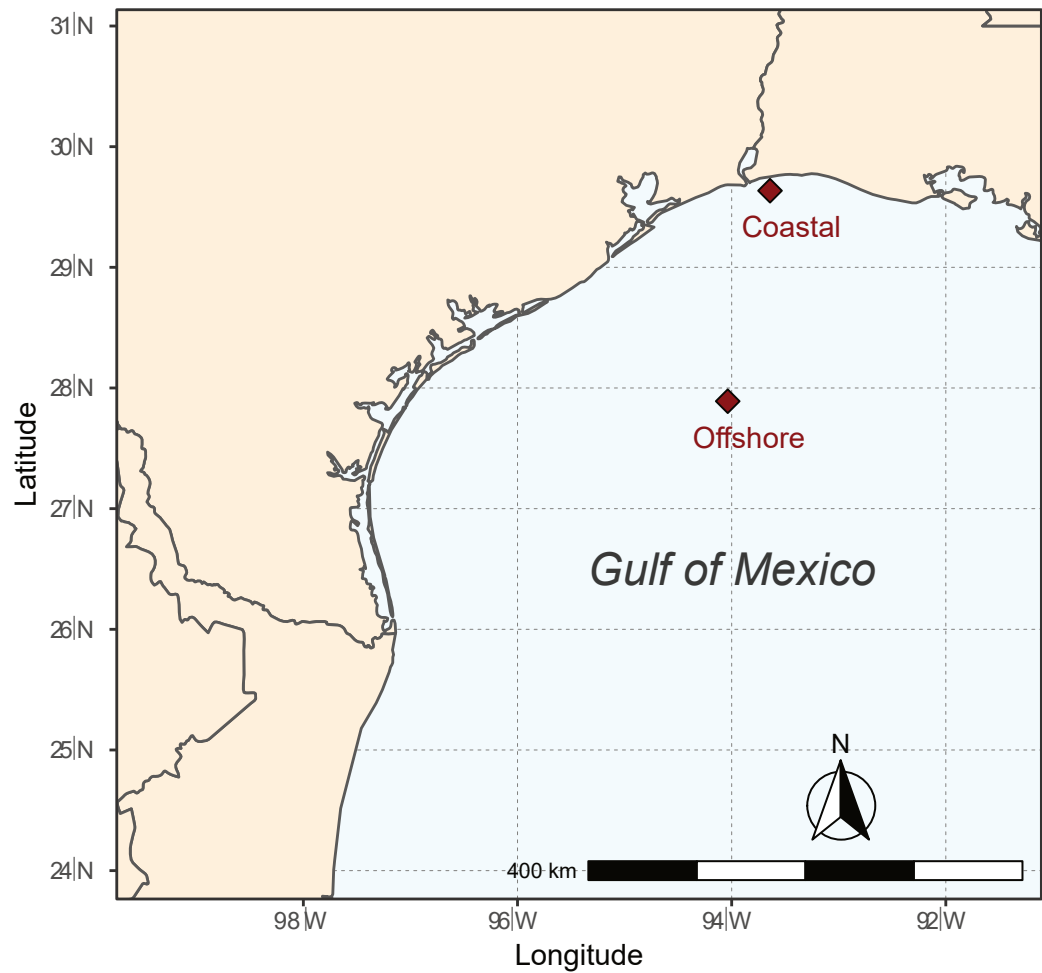

Supplement: FIG S1 [file mSystems.00668-20-sf001.pdf]

# Mesocosm Cell counts

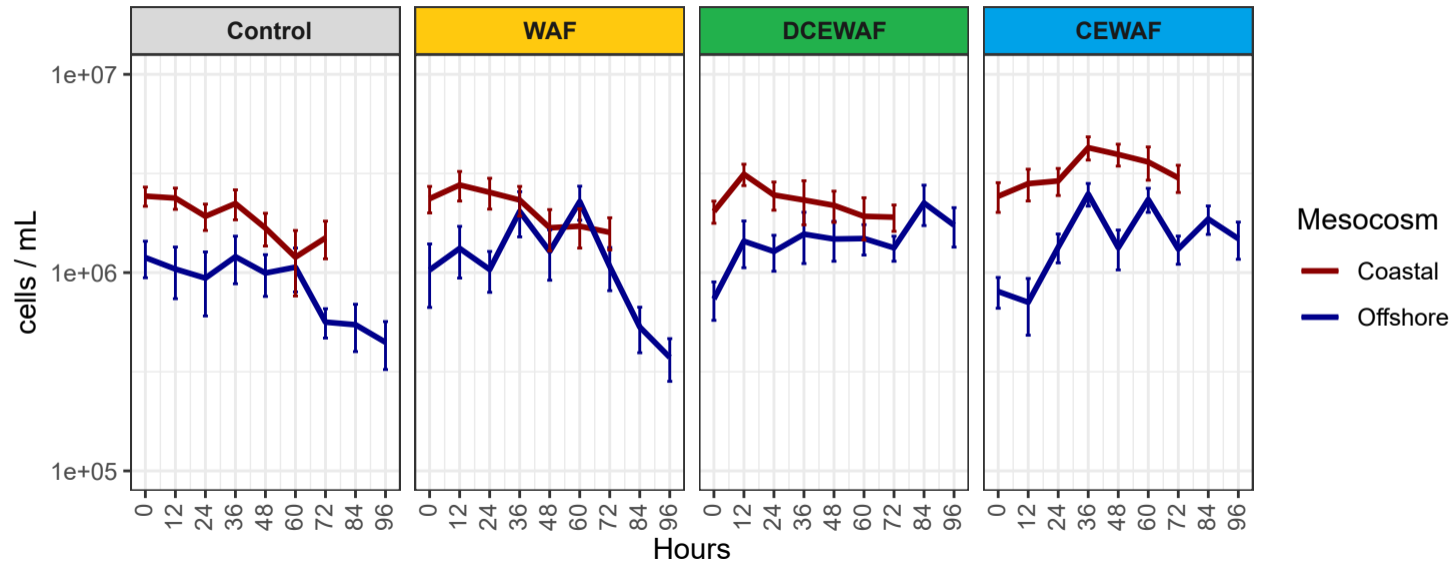

Supplement: FIG S2 [file mSystems.00668-20-sf002.pdf]

Bray-Curtis NMDS (Stress: 0.092)

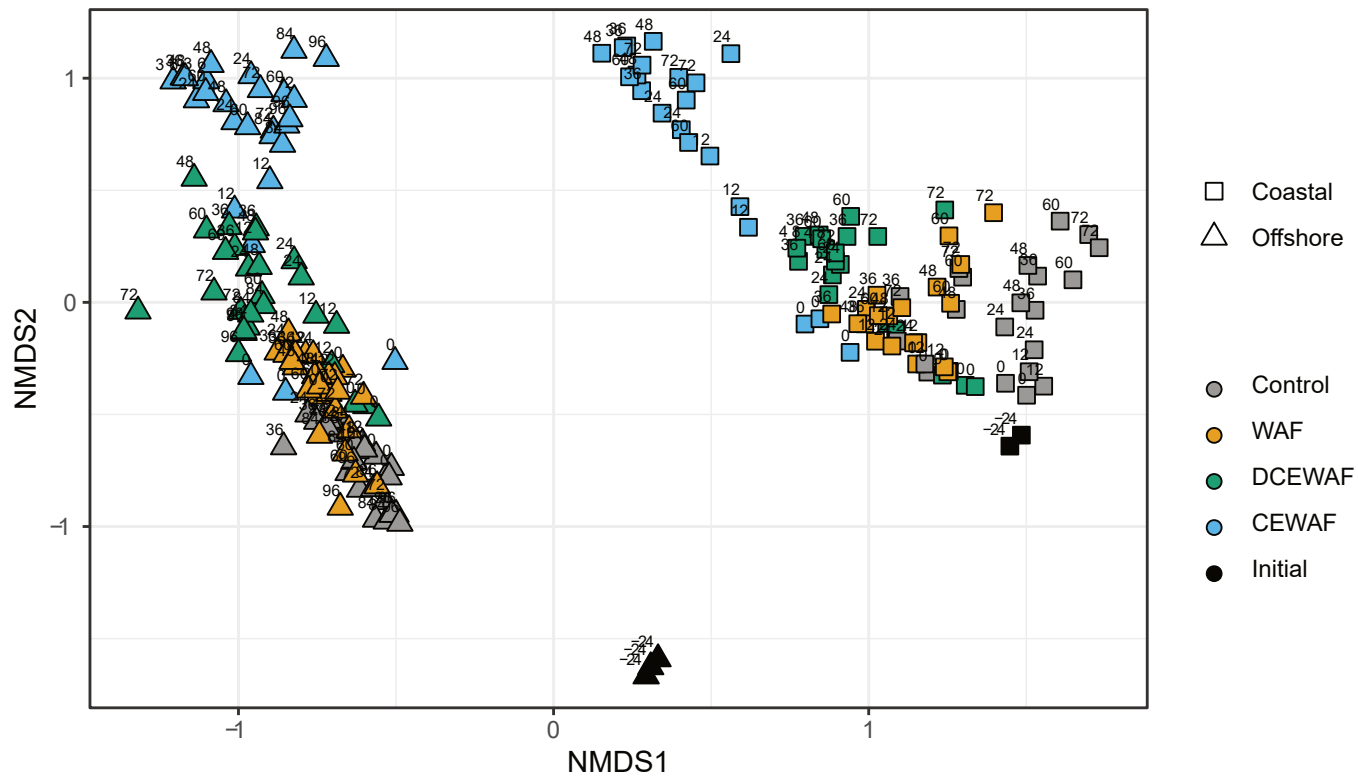

Supplement: FIG S3 [file mSystems.00668-20-sf003.pdf]

**A** Coastal (Stress: 0.094)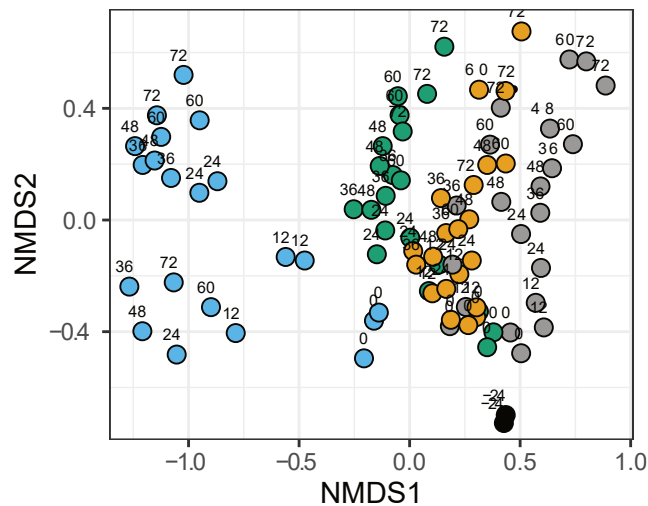**B**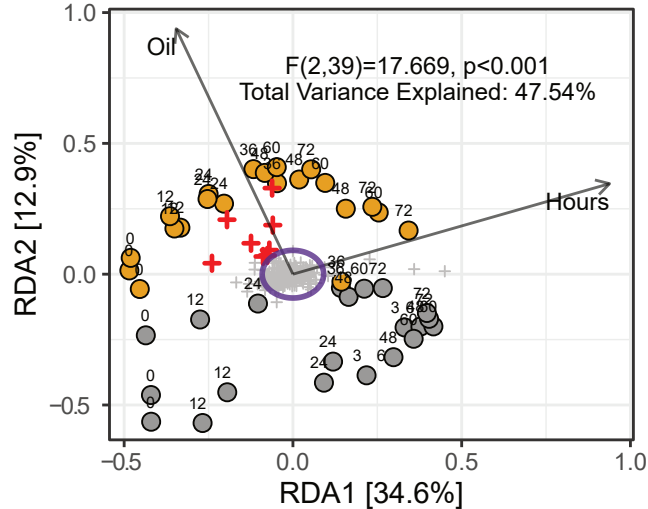**C**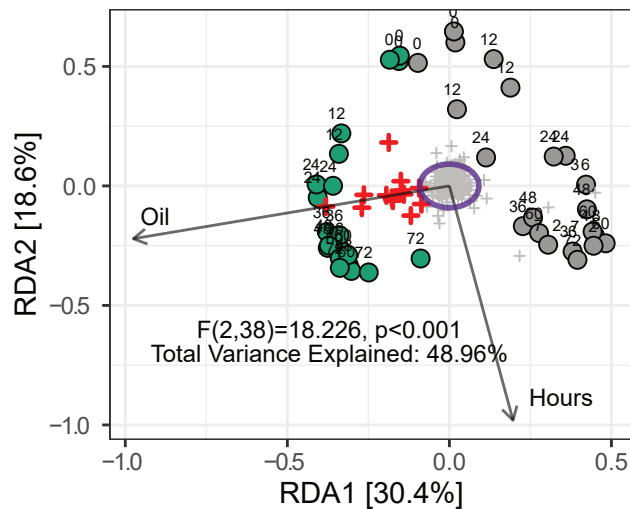**D**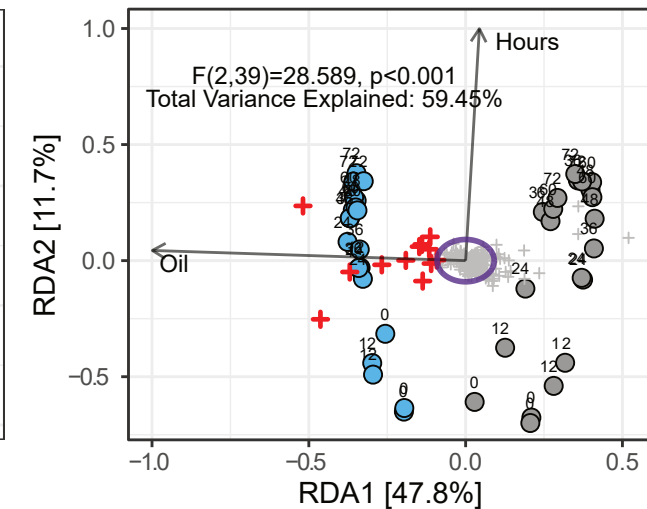**E** Offshore (Stress: 0.082)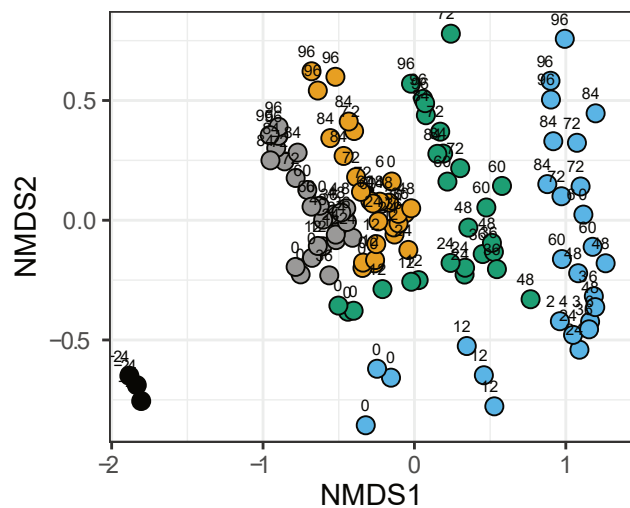**F**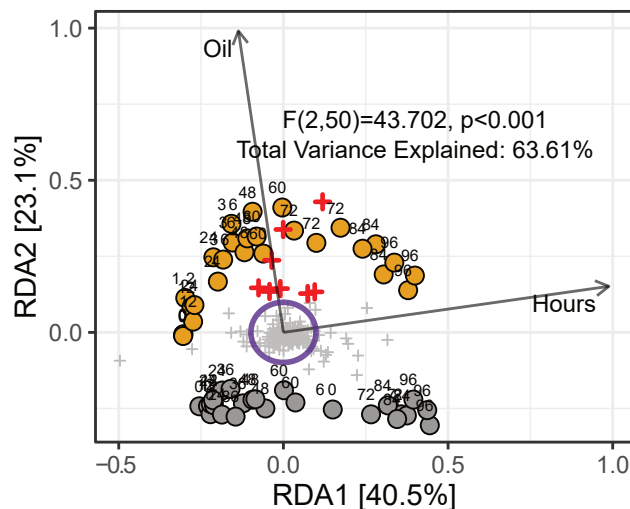**G**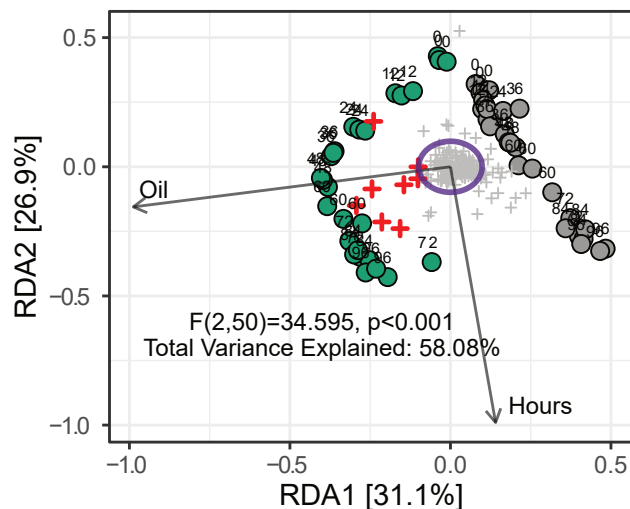**H**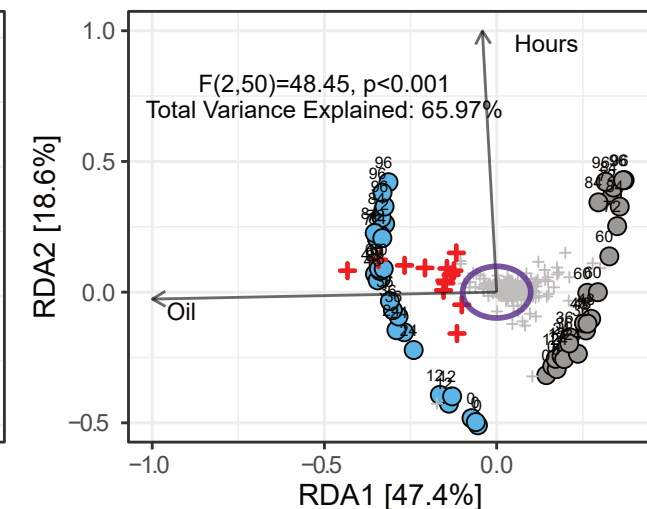

● Initial    ● Control    ● WAF    ● DCEWAF    ● CEWAF

Supplement: FIG S4 [file mSystems.00668-20-sf004.pdf]

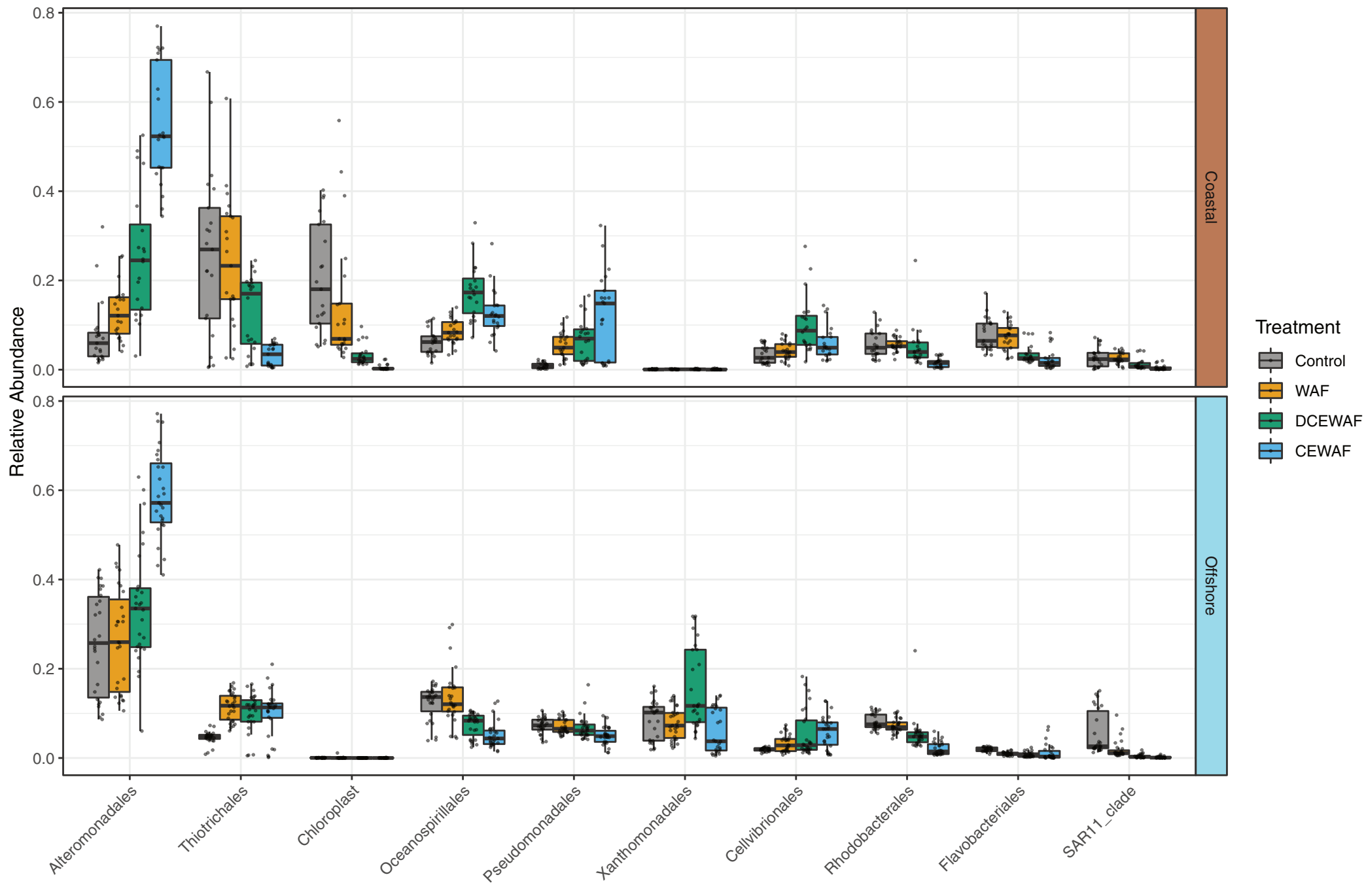

Supplement: FIG S6 [file mSystems.00668-20-sf006.pdf]

**A****Coastal**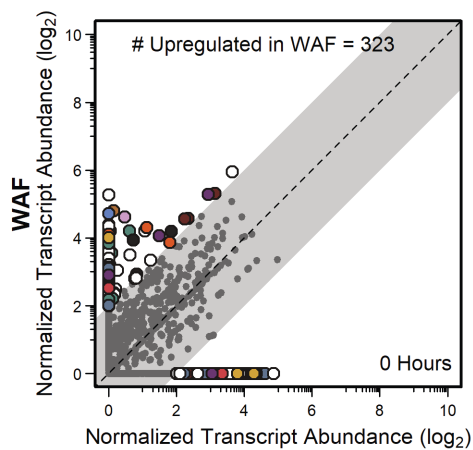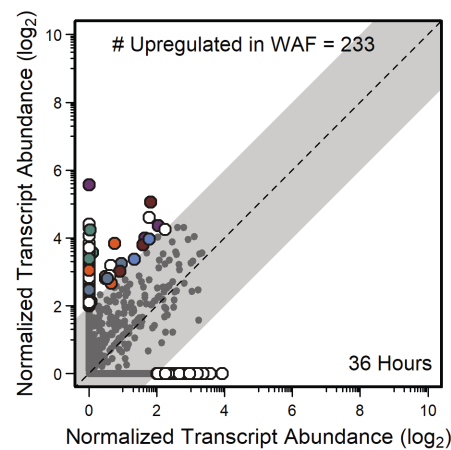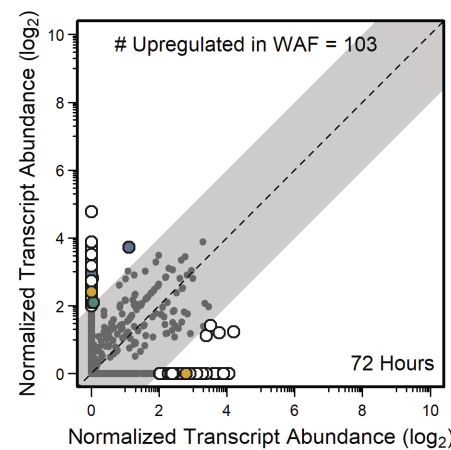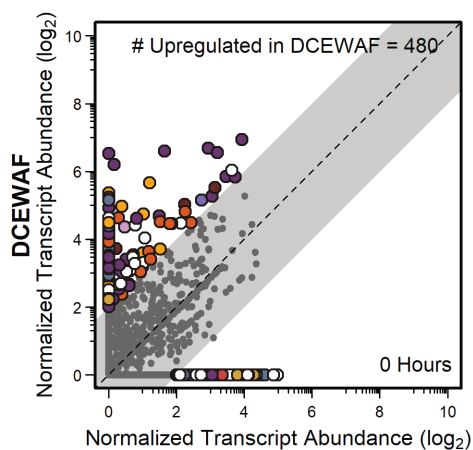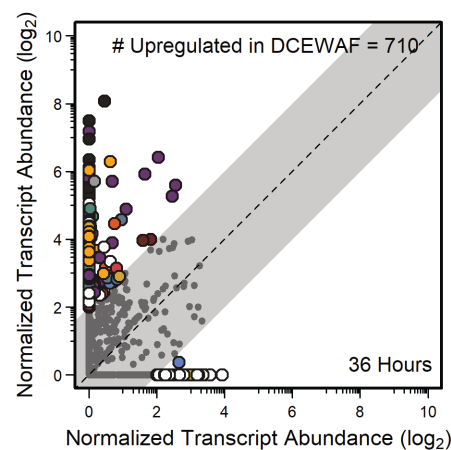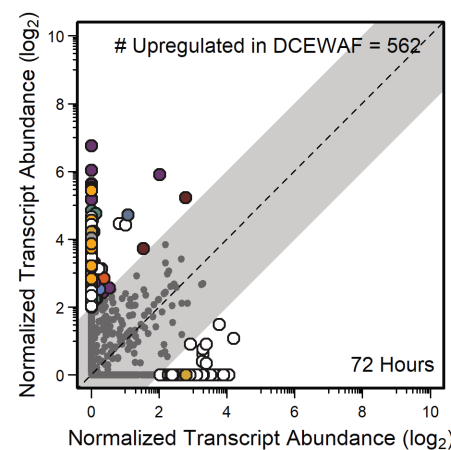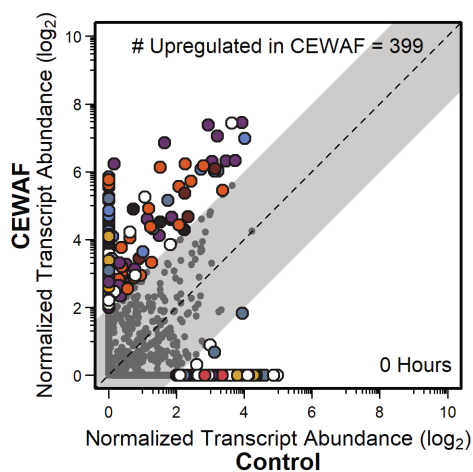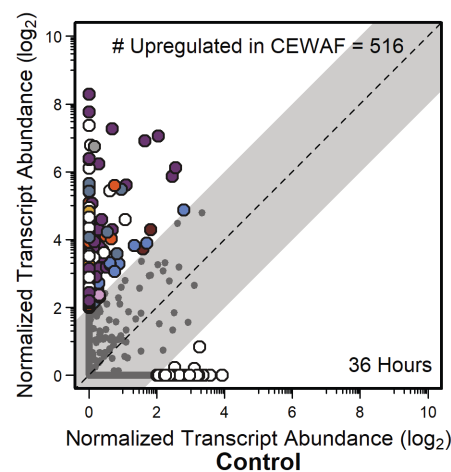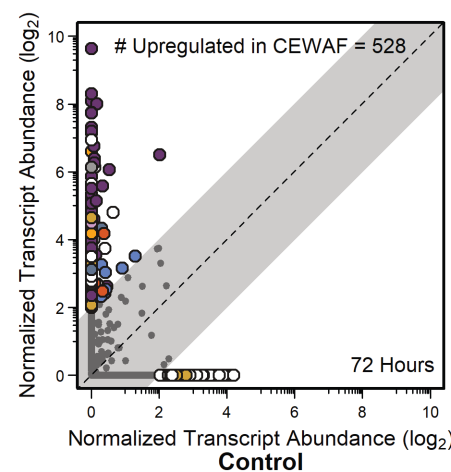**B****Offshore**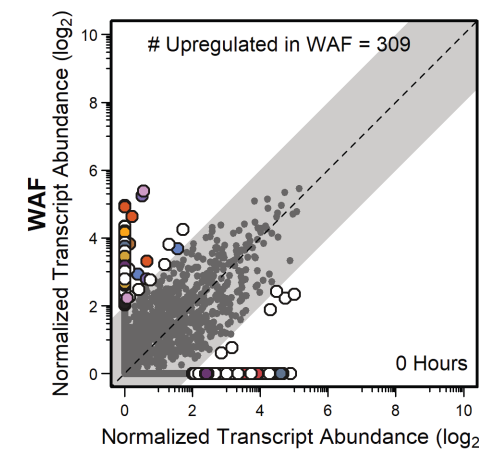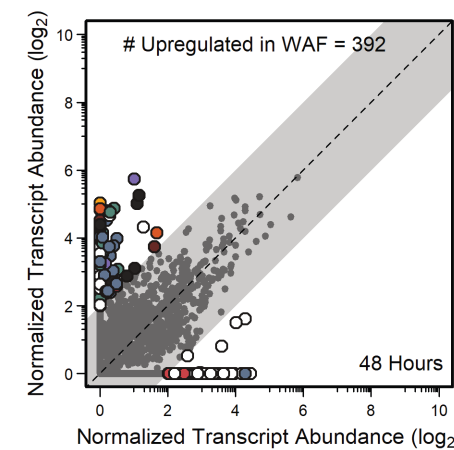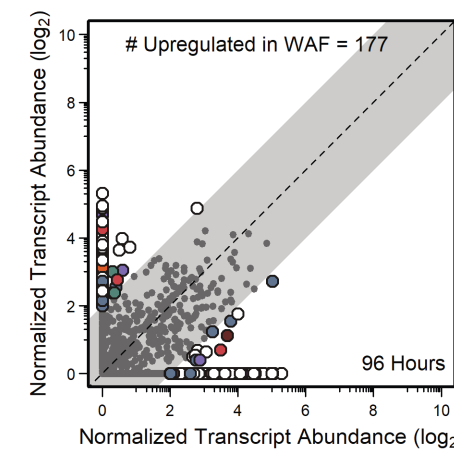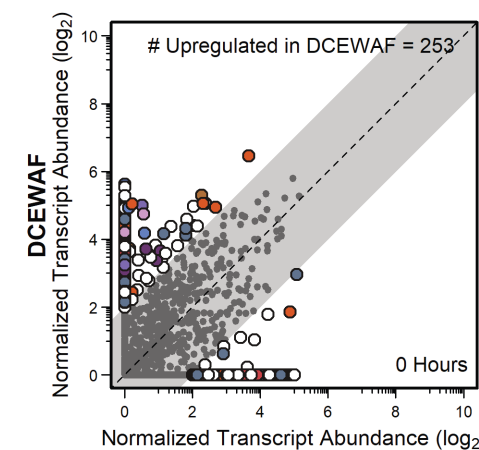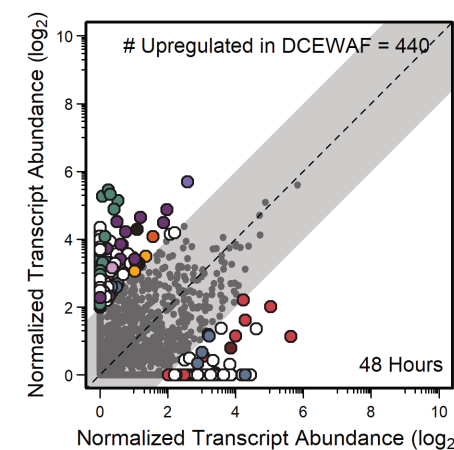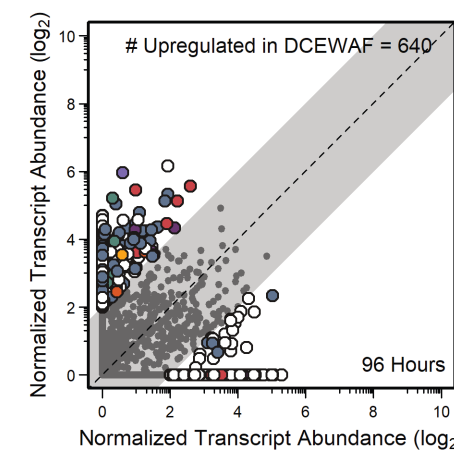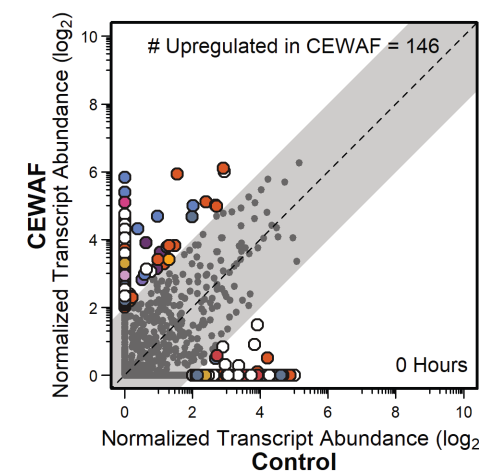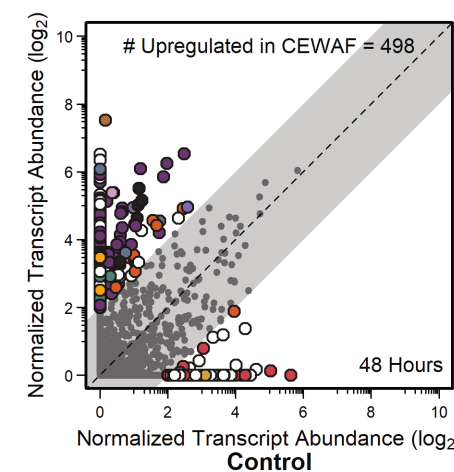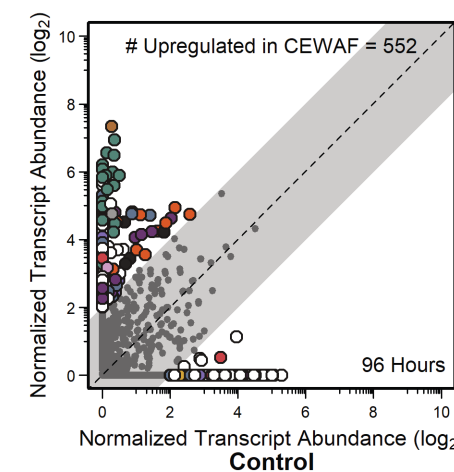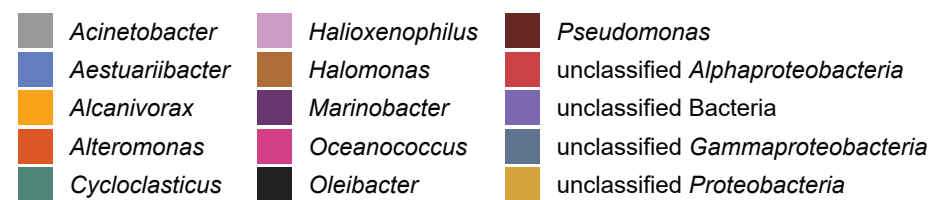

Supplement: FIG S7 [file mSystems.00668-20-sf007.pdf]

# of ASVs

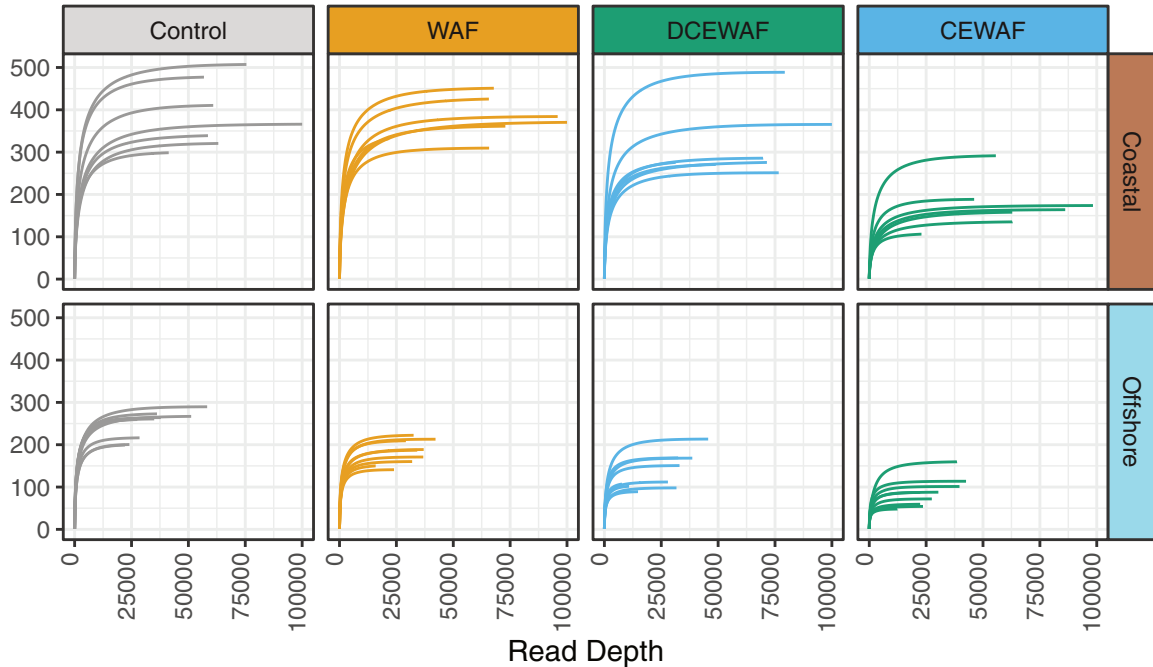

Supplement: FIG S9 [file mSystems.00668-20-sf009.pdf]
