# Supplementary material for: Niche Partitioning between Coastal and Offshore Shelf Waters Results in Differential Expression of Alkane and Polycyclic Aromatic Hydrocarbon Catabolic Pathways
Source: mSystems. 2020 Aug 25;5(4):e00668-20. doi: 10.1128/mSystems.00668-20 (PMC7449609; doi:10.1128/mSystems.00668-20)

Hydrocarbon degradation gene expression levels did not vary substantially between treatment

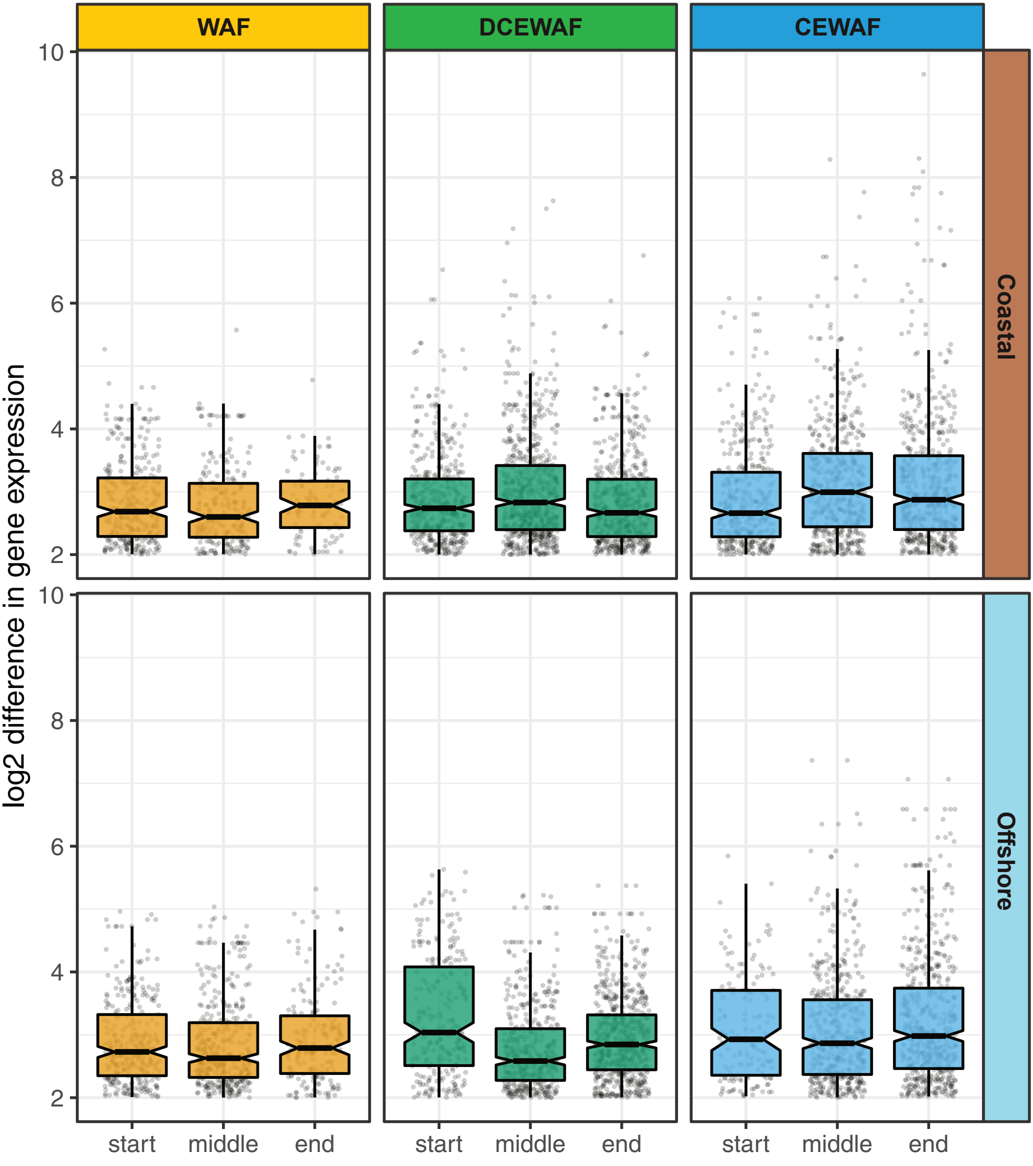

Supplement: FIG S8 [file mSystems.00668-20-sf008.pdf]
