# Supplementary material for: Niche Partitioning between Coastal and Offshore Shelf Waters Results in Differential Expression of Alkane and Polycyclic Aromatic Hydrocarbon Catabolic Pathways
Source: mSystems. 2020 Aug 25;5(4):e00668-20. doi: 10.1128/mSystems.00668-20 (PMC7449609; doi:10.1128/mSystems.00668-20)
Supplement: FIG S5 [file mSystems.00668-20-sf005.pdf]

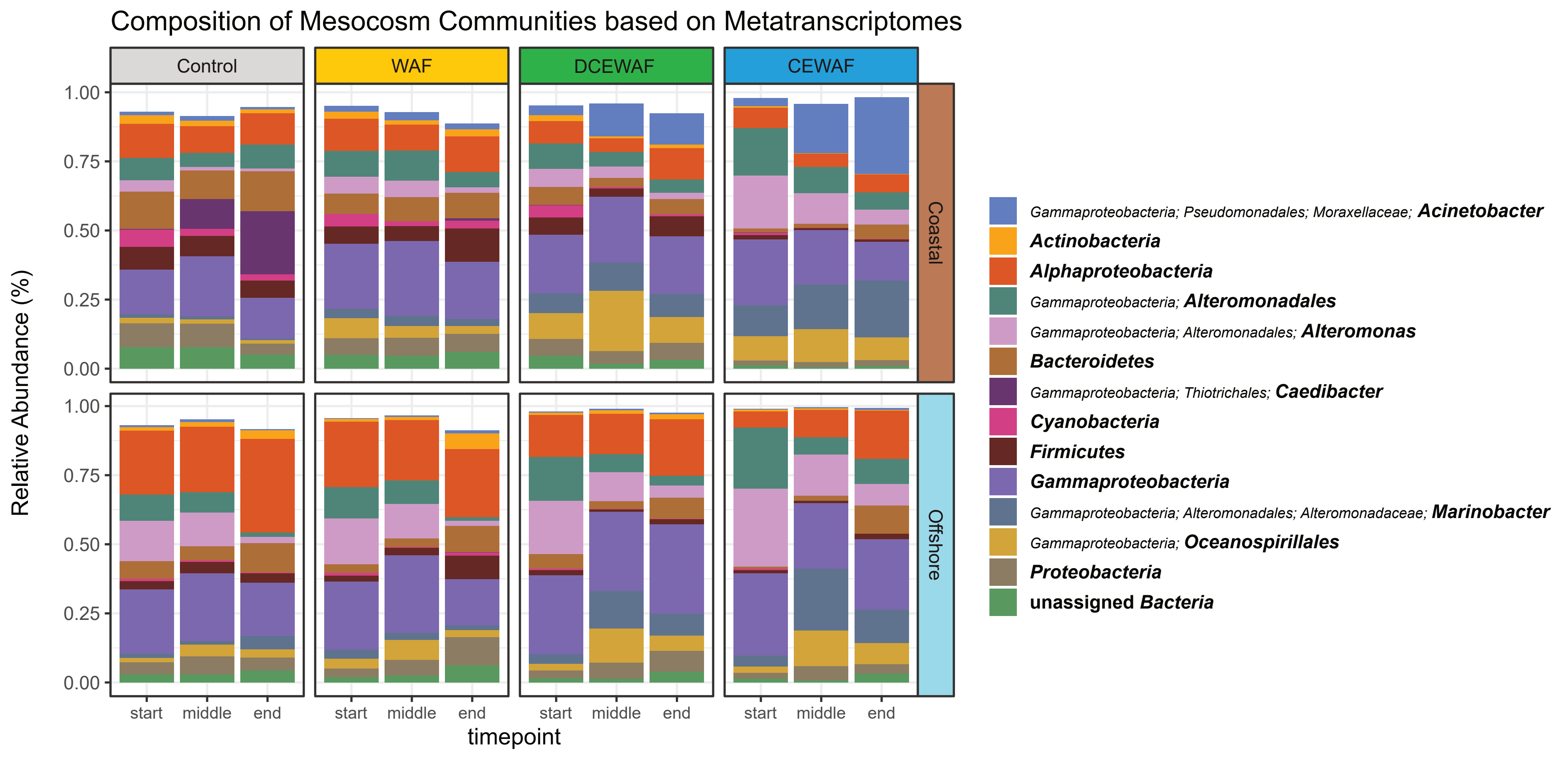

Composition of Mesocosm Communities based on Metatranscriptomes
Control
WAF
DCEWAF
CEWAF
Coastal
Offshore
Relative Abundance (%)
1.00
0.75
0.50
0.25
0.00
start
middle
end
timepoint
Gammaproteobacteria; Pseudomonadales; Moraxellaceae; Acinetobacter
Actinobacteria
Alphaproteobacteria
Gammaproteobacteria; Alteromonadales
Gammaproteobacteria; Alteromonadales; Alteromonas
Bacteroidetes
Gammaproteobacteria; Thiotrichales; Caedibacter
Cyanobacteria
Firmicutes
Gammaproteobacteria
Gammaproteobacteria; Alteromonadales; Alteromonadaceae; Marinobacter
Gammaproteobacteria; Oceanospirillales
Proteobacteria
unassigned Bacteria
